# The Impact on the Quality of Life Among Newly Diagnosed Children With Coeliac Disease in Malta: A Child Versus Parent Perspective

**DOI:** 10.7759/cureus.76133

**Published:** 2024-12-21

**Authors:** Sophie N Hackenbruch, Nadine A De Battista, Sarah G Grech, Anne M Grima

**Affiliations:** 1 Paediatrics, Mater Dei Hospital, Msida, MLT; 2 Genetics, Mater Dei Hospital, Msida, MLT; 3 Ophthalmology, Mater Dei Hospital, Msida, MLT

**Keywords:** children, coeliac disease, gluten, parent child relationship, pediatric gastroenterology, quality of life, screening

## Abstract

Background and objective

Coeliac disease (CD) is an autoimmune condition that is managed by following a strict lifelong gluten-free diet. Its incidence is rising, and no cure is currently available. CD in children has a significant impact on both patients and their caregivers as they adapt to a new lifestyle. Tools to assess the quality of life (QoL) of children with chronic conditions can shed some light on the disease burden on these families. This study aimed to evaluate the impact on the QoL for newly diagnosed children with CD, considering and comparing both the child’s and caregivers’ perspectives. It covers various themes including physical and psychological health, the impact on education, and the financial burden on affected families. We sought to evaluate the multidimensional impact of CD on the QoL of newly diagnosed children and their caregivers in Malta and to identify key disparities between child and caregiver perceptions. Different aspects of QoL were assessed, such as financial matters, emotional stress, and physical activity, which were quantified using the standardized KIDSCREEN and CDDUX questionnaire. The perspectives of different patient groups (classified by gender, symptomatology, and age) were compared, providing insights into the differences, which may help refine the management.

Methods

This was a retrospective study, collecting data from May 2022 to January 2023. All children under 16 years of age diagnosed with coeliac disease from January 2020 until January 2022 on the Maltese islands, were included in the study. Patients older than 16 years of age or those who had deceased were excluded from the study. The survey comprised the KIDSCREEN-52 and CDDUX questionnaires to assess the QoL.

Results

A total of 268 children fit the criteria for inclusion, with 134 (50%) children and 134 (50%) matched parent responses. CD was more common among females - 85 (63%) compared to 45 (34%) boys - and children living in the northern region of Malta: 44 (33%). There was no statistically significant difference in QoL when comparing the perspectives of parents vs. children, adolescents vs. young children, boys vs. girls, and asymptomatic vs. symptomatic patients. No significant financial burden was highlighted by the participants.

Conclusions

The QoL of children with CD in Malta is satisfactory. The perspectives of parents and children on the condition's impact on QoL mostly align, with differences mainly noted regarding the awareness of emotional and social struggles. Our findings suggest that an early CD diagnosis, irrespective of the symptomatology, age, or gender, does not negatively impact the QoL of Maltese children. The study also highlights that despite overall good health, providing adequate psychological, financial, and social support for these children and their families is important to achieve positive outcomes, together with raising further public awareness about this condition.

## Introduction

Coeliac disease (CD) is a chronic autoimmune gastrointestinal (GI) condition that occurs in genetically predisposed individuals and is characterized by common GI symptoms such as abdominal pain, abdominal distension, and Diarrhea [1]. The global prevalence of CD in children is 0.7% [2], and it has been steadily on the rise [3]. In children, this diagnosis may be elusive as it may present with vague symptoms such as failure to thrive or skin rashes. The investigation and diagnosis of CD in children have been researched at length and European guidelines are available to guide clinicians in making this diagnosis. Screening programs for CD have also revealed a new patient group of asymptomatic children [4]. The introduction of screening programs has led to earlier identification and increased incidence rates of CD [5]. It is still disputed whether the benefits of an early diagnosis, especially in asymptomatic patients, outweigh the challenge of subjecting a child to a strict lifelong dietary treatment [6]. No curative treatment is currently available, and despite a strict gluten-free diet, some patients still experience duodenal injury [1].

It is well known that chronic conditions impact the quality of life (QoL) for both the affected individuals, and, especially in the case of children, their caregivers. Currently, the ICT Tools for the Diagnosis of Autoimmune Diseases in the Mediterranean Area (ITAMA), an INTERREG V-A Italia-Malta Cooperation Project funded by the European Regional Development Fund, is carrying out a nationwide CD screening program among children. The project aims to raise awareness, validate the point-of-care screening test, and reduce the cost of the disease burden caused by a CD diagnosis. Primarily, this is a research project to assess the effectiveness of the point-of-care coeliac test to reduce the need for invasive testing in CD [7].

KIDSCREEN and CDDUX are two standardized tools used to assess QoL in children diagnosed with CD. The KIDSCREEN questionnaire has been translated into several languages. It assesses both the caregiver's and the child’s view of the affected child’s QoL. The KIDSCREEN-52 is the latest generic questionnaire in use. It is a tool that can assess QOL in children aged 8-18 years [8]. CDDUX was developed in 2009 as the first questionnaire to evaluate QOL among children aged 8-18 years with CD. It consists of a Likert scale of faces with different emotions for the child to choose from when asked about different aspects of living with CD [9]. There are scarce studies investigating similarities and differences in perspectives between children with CD and their caregivers regarding the impact on QoL. This study is the first of its kind to address the impact of a CD diagnosis on the QoL in the Maltese population. It also aims to shed light on epidemiological data for different genders, age groups, geographical locations, and symptomatology.

## Materials and methods

We employed a retrospective design to conduct this study, collecting data from May 2022 till January 2023. All children under 16 years of age, at the time of diagnosis, who had been diagnosed with CD from January 2020 until January 2022, were included in the study. These two years constituted the first years when nationwide screening was implemented on the Maltese islands. Children from all genders, social backgrounds, nationalities, and medical backgrounds were included. Patients over 18 years of age or deceased patients were excluded from the study. We aimed to ensure a sample size representative of children nationwide diagnosed with CD over a two-year period when the screening was carried out for the first time, enabling a high yield. The list of children was compiled using in-house hospital records, the National Medicine Entitlement system, and the National Coeliac Disease Screening Database (formed as part of the ongoing ITAMA project screening children between 6 and 16 years of age for CD). Demographic data and symptomatology were collected using in-house records. Data were collected using an anonymous online survey, which was distributed to the children’s parents after obtaining consent for participation.

The online survey was distributed using a study-specific email address to which only the researchers were given access; after the initial email, two reminders, one month apart, were sent to all participants. The questionnaire was distributed one year post-diagnosis. Questions in the survey were based on the KIDSCREEN and CDDUX questionnaires and explored the following themes: physical activities and health, emotional wellbeing, attitude towards physical appearance, leisure time, relationship with parent/carer(s), financial burden, relationships with peers, school performance and education, bullying, attitudes towards adherence to a gluten-free diet, and attitudes towards sharing diagnosis with others. Parents and children were advised to respond independently and confine their responses to their respective sections. Data were stored anonymously on a password-protected Excel sheet only accessible to the researchers. Ethical approval was obtained from the University of Malta Ethics Committee. 

The survey was split into two parts with the first directed towards the parent(s) and the second part directed towards the child in question. Answers were then compiled and analyzed using Microsoft Excel software. The Mann-Whitney U test was used for statistical analysis of different subject groups, to compare caregiver vs. child perspectives as well as those between children in different age groups, with different symptomatology, etc. A p-value <0.05 was considered statistically significant.

## Results

Demographic data

A total of 268 children fit the criteria for inclusion in the study, with 134 (50%) children and 134 (50%) parent responses. The demographic data are presented in Table 1. The results from the KIDSCREEN and CDDUX questionnaires were grouped according to their respective themes.

**Table 1:** Demographic data

Characteristics	N	%
Age, years		
04-Jul	23	17.16
08-Nov	47	35.07
Dec-15	50	37.31
16-18	14	10.45
Locality		
Central Region	24	17.91
Gozo	9	6.72
Northern Region	44	32.84
Southeastern	25	18.66
Southern	32	23.88
Gender		
Male	49	36.57
Female	85	63.43

Physical activities and health

**Figure 1:**
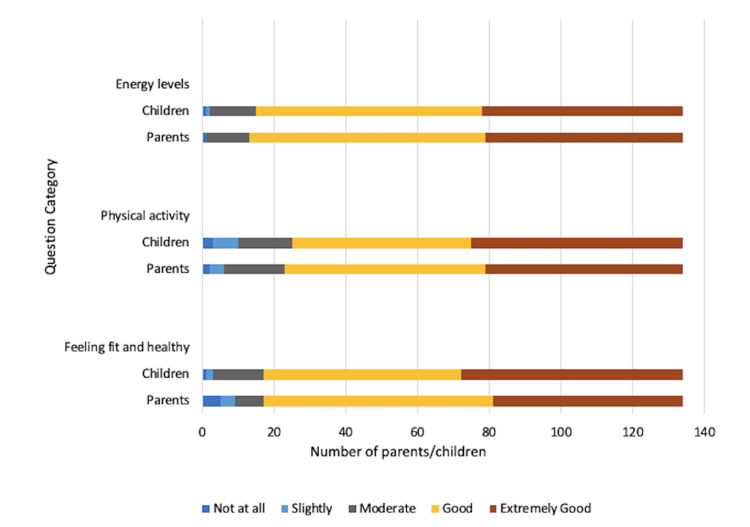
Impact of coeliac disease diagnosis on the child’s physical activities and health

Figure 1 demonstrates responses to questions covering the impact of a CD diagnosis on the child’s physical activities and health, from both the child’s and the parent’s perspectives. Sixty-five (47.5%) children in the cohort reported feeling fit and healthy despite their diagnosis, with 45 (40.8%) parents confirming this. The majority (both children and parents) also reported good to extremely good energy levels.

Emotional well-being

**Figure 2:**
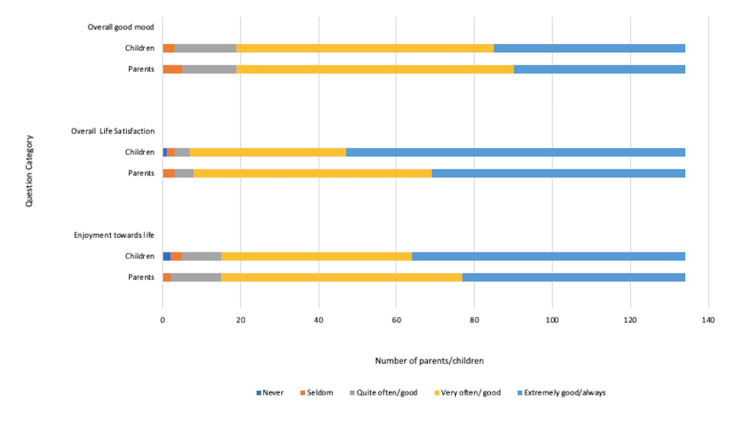
Impact of coeliac disease diagnosis on the child’s feelings and emotional well-being

Figure 2 depicts the responses to questions covering the impact of a CD diagnosis on emotional well-being. The majority of the children and parents reported overall good mood and feelings of enjoyment towards life despite the diagnosis. Interestingly, 61 (45.5%) parents reported good overall child life satisfaction, in contrast to the 40 (29.8%) reported by the children.

General mood

**Figure 3:**
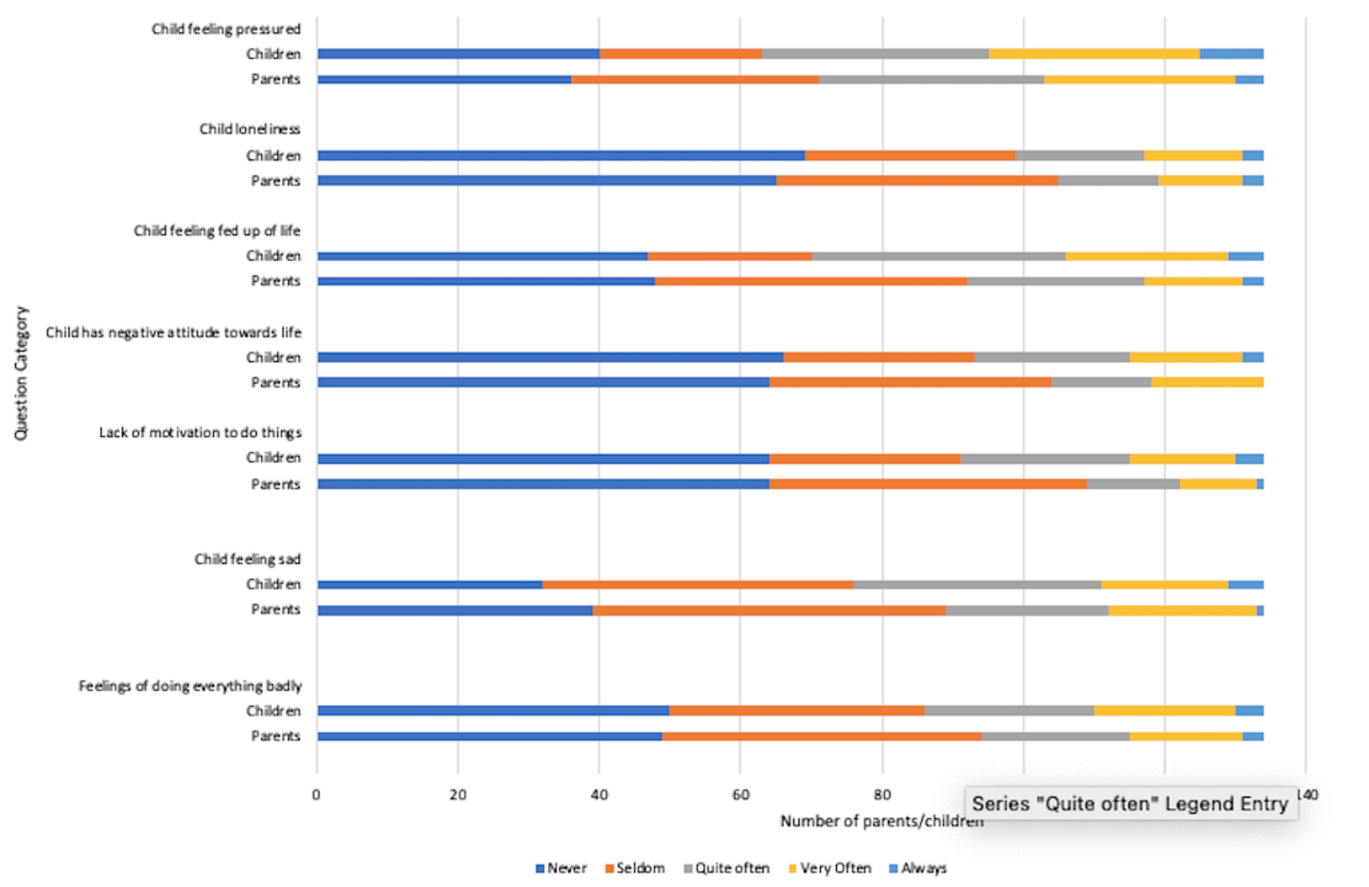
Impact of coeliac disease diagnosis on the child’s general mood

The majority of children (and their parents) denied feelings of sadness, loneliness, or lack of motivation to do things. However, 35 (26%) children reported feeling sad “quite often” with 36 (26%) reporting “quite often being fed up with life”. A comparison of child vs. parent responses highlighted that parents seemed to underestimate their children’s feelings of loneliness, pressure, and negative attitude toward life (Figure 3).

Attitudes toward physical appearance

**Figure 4:**
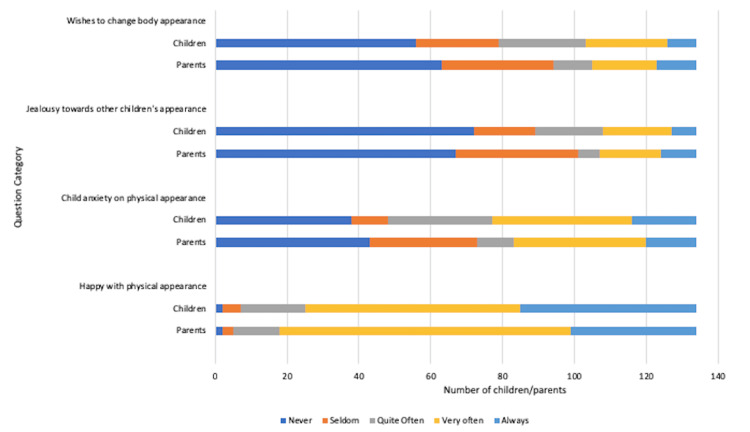
Impact of coeliac disease diagnosis on attitudes towards physical appearance

Questions covering attitudes towards physical appearance showed that up to 60 (44.8%) children in the cohort were happy with their appearance, with 72 (53.7%) showing no jealousy towards others and 59 (43%) never expressing wishes to change their bodies. Parental views mostly aligned with these responses. However, 86 (64.1%) children admitted to feeling anxious about their looks often, with 18 (13.4%) reporting constant anxiety. Once again, parental views seemed to underestimate children’s negative views towards their physical appearance, as seen in Figure 4.

Leisure time

**Figure 5:**
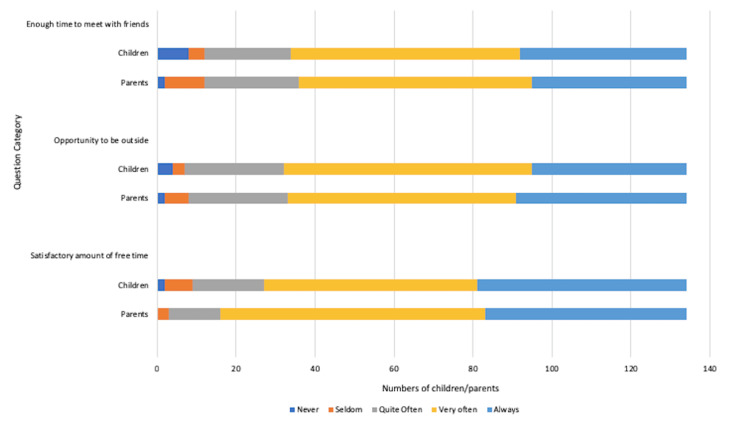
Impact of coeliac disease diagnosis on free time

More than 125 (50%) children in the cohort felt that their CD diagnosis did not affect their leisure time, with a satisfactory amount of time dedicated to relaxation activities and meeting up with friends. Parental views overall aligned with those of their children, as depicted in Figure 5.

Family and home life

**Figure 6:**
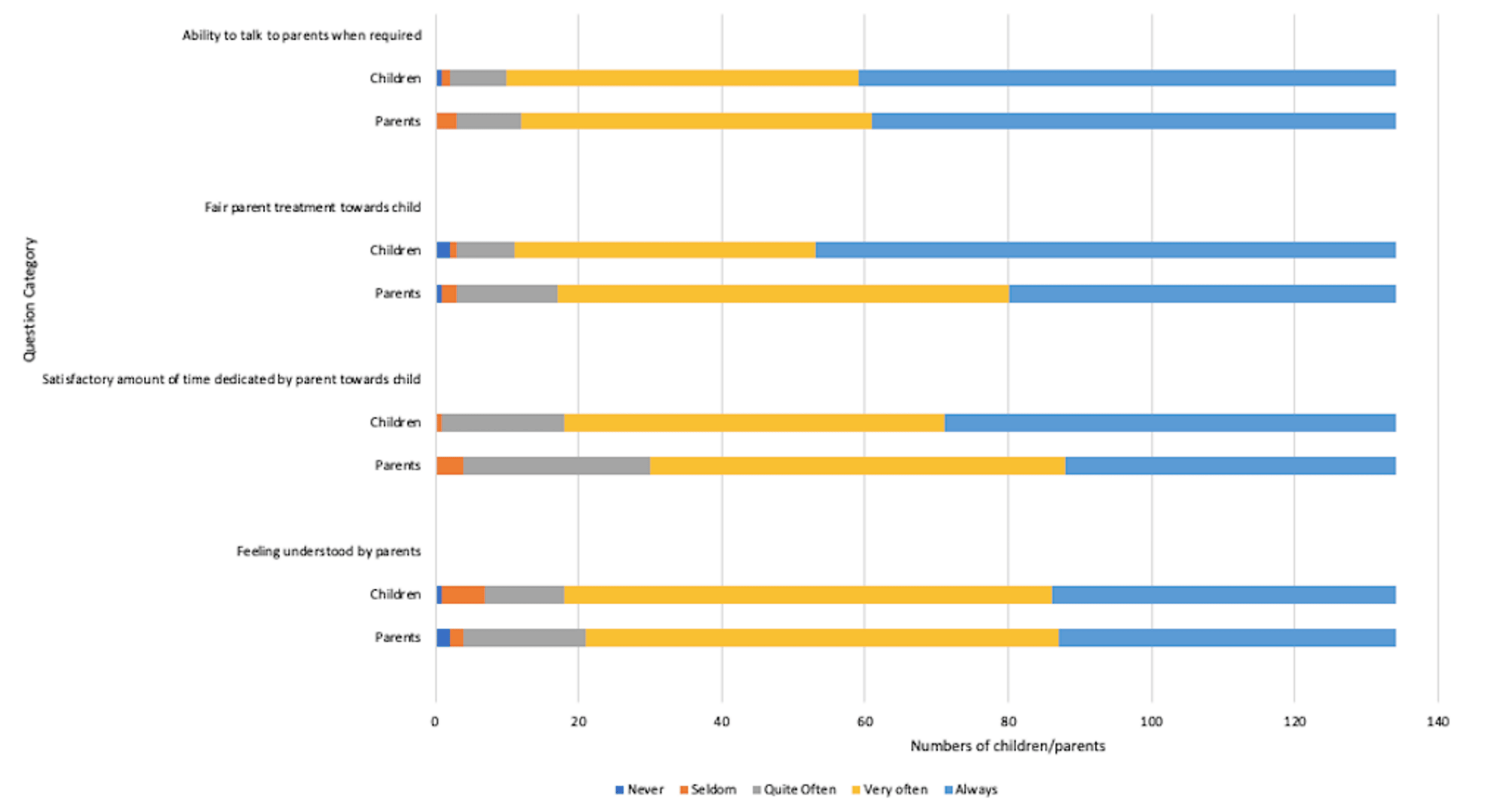
Impact of coeliac disease diagnosis on family and home life

Family, home life, and the child-parent relationship were also covered in our study, and the results are depicted in Figure 6. More than 127 (80%) children in the cohort reported that they felt well-understood by their parents and were able to talk to them when the need was felt. More than 133 (95%) children and parents reported fair parent treatment towards the child with a satisfactory amount of time dedicated to the child’s needs.

Financial matters

**Figure 7:**
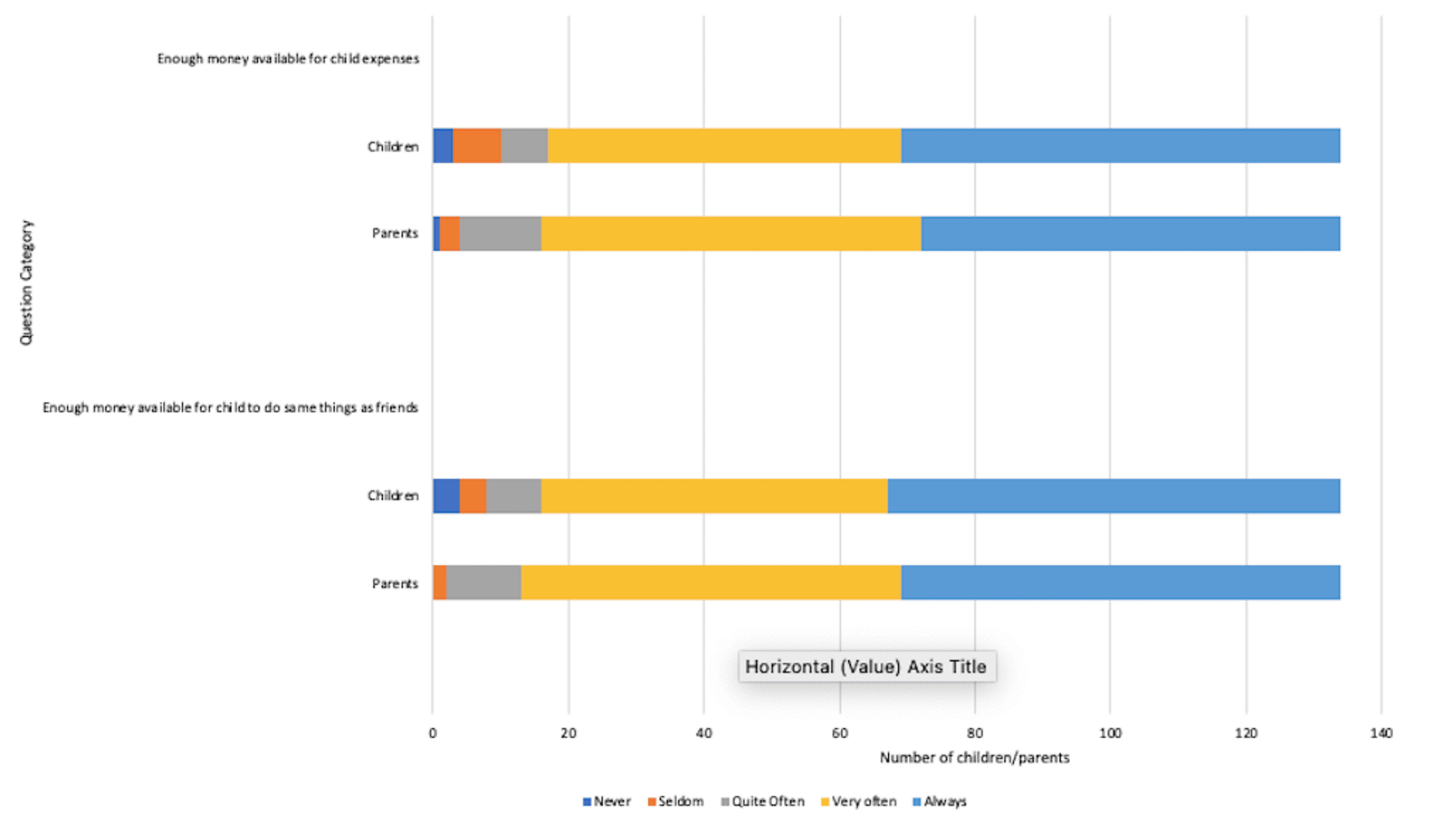
Impact of coeliac disease diagnosis on financial matters

Questions relating to financial matters (results presented in Figure 7) revealed that 132 (99%) parents and 126 (94%) children felt that adequate money was available to cater for their child’s expenses with enough money available to do the same things as their friends [parents: 129 (96%), children: 124 (93%)].

Friendships

**Figure 8:**
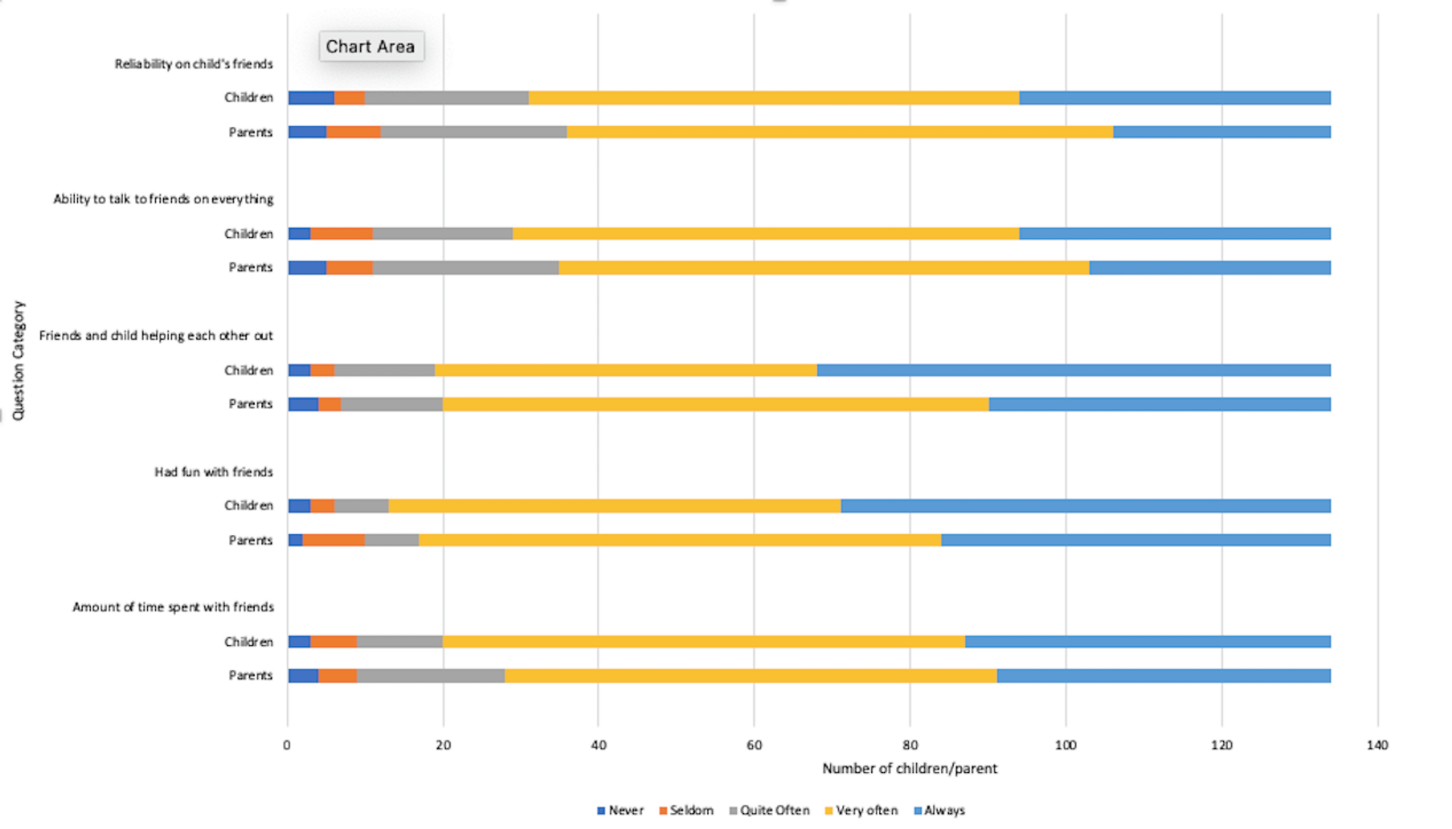
Impact of coeliac disease diagnosis on friendships

Most children and parents reported maintaining good friendships, with more than 125 (85%) children feeling that adequate time is still spent with their friends despite their diagnosis. More than 127 (94%) parents felt that the children and their friends help each other often, with 105 (79%) children feeling that they can talk to their friends on all subject matters including CD. Parent views aligned, as seen in Figure 8.

School and learning

**Figure 9:**
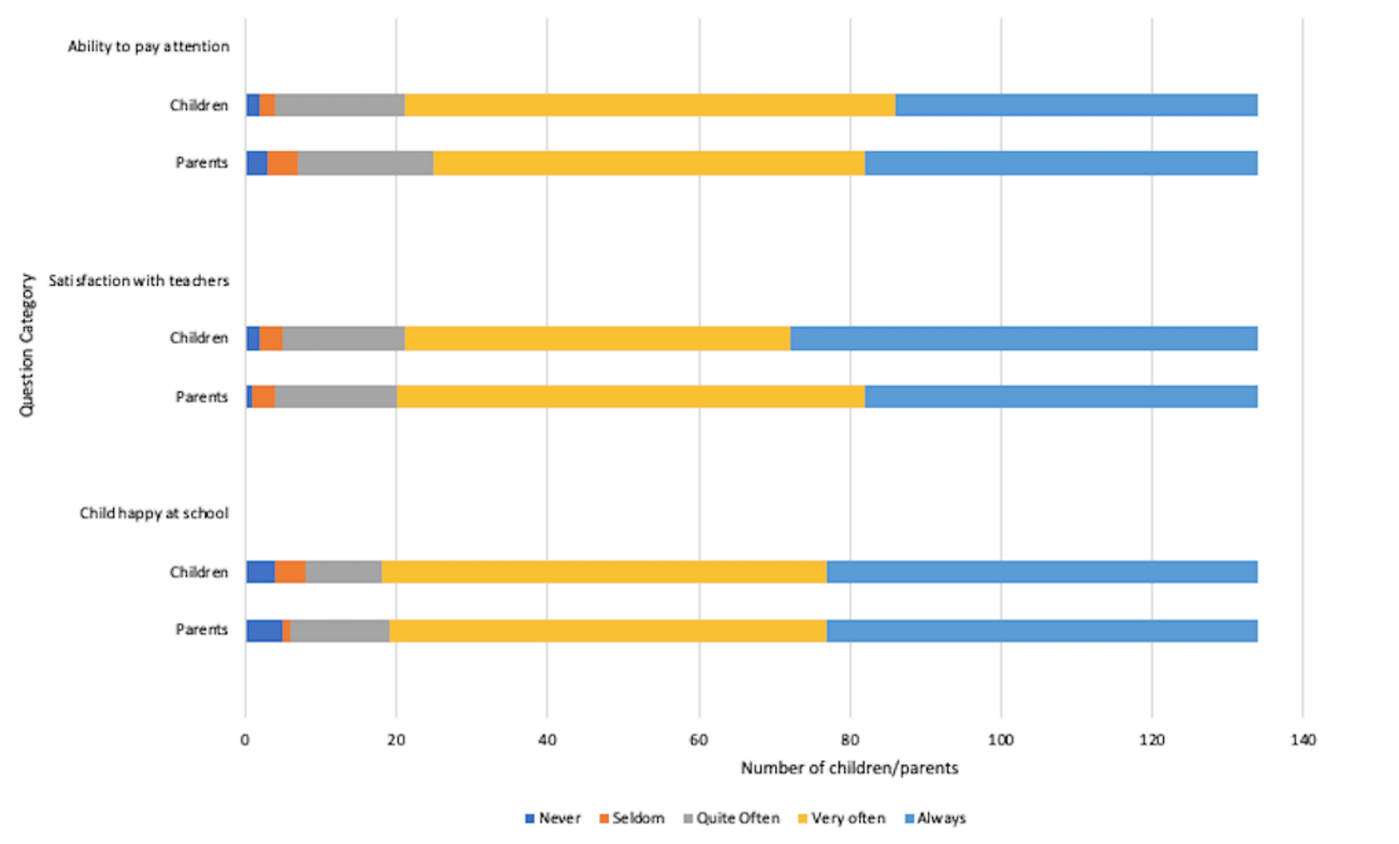
Impact of coeliac disease diagnosis on school and learning

More than 116 (86.7%) children in the cohort felt happy at school with 129 (96%) feeling satisfied with their teachers. Of note, 113 (84%) children felt that their CD diagnosis did not affect their attention span during learning. As shown in Figure 9, the parental views once again seemed to align with those of their children.

Bullying

**Figure 10:**
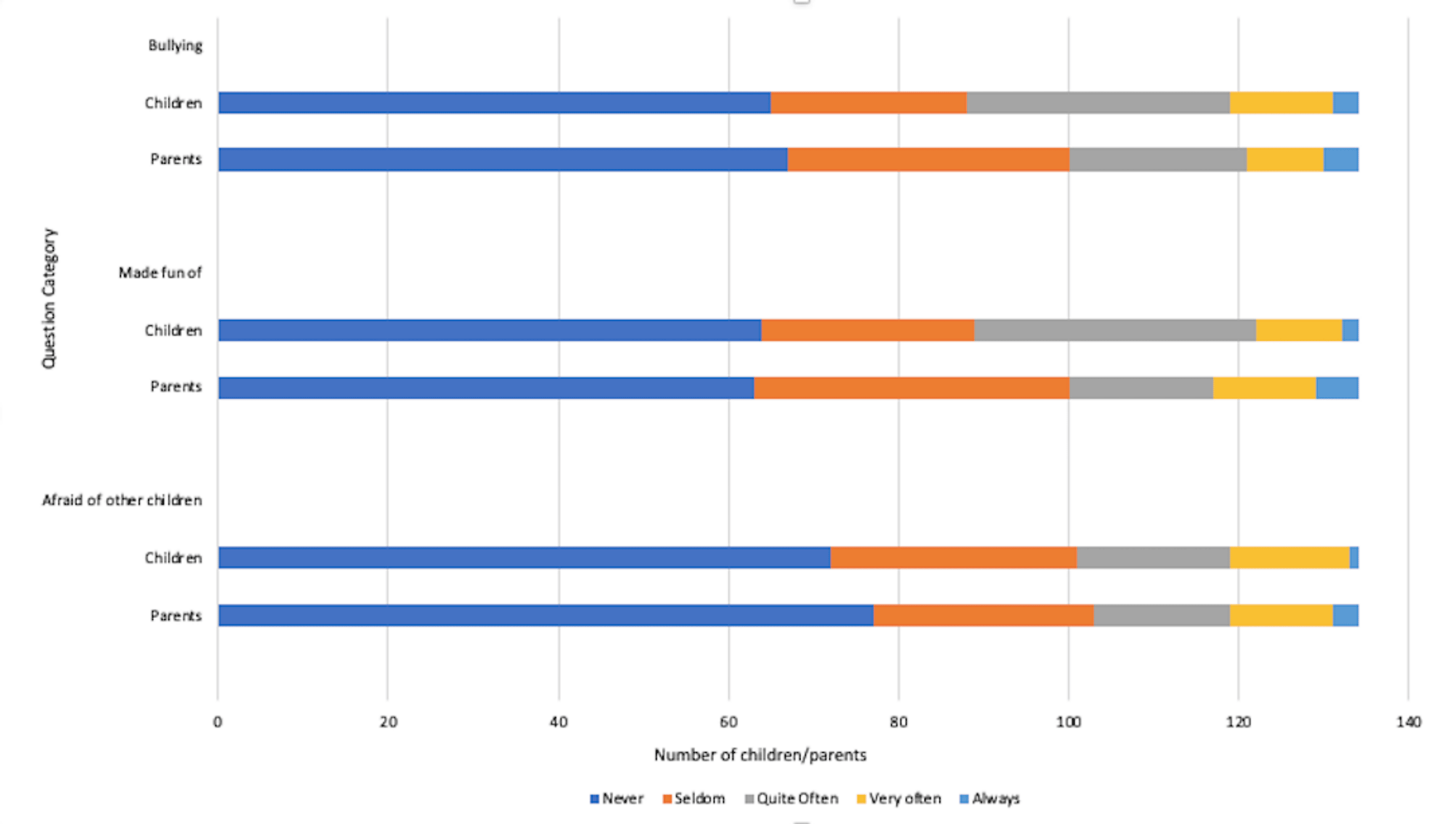
Impact of coeliac disease diagnosis on peer relationships

Peer relationships and the topic of bullying were also explored in this study, with most children denying bullying or fear of other children. However, 43 (34%) of the study population did report bullying happening quite often, though not necessarily related to their medical diagnosis. Interestingly parents seemed to underestimate children’s exposure to bullying [30 (24.8%) parents vs. 43 (34%) children], as illustrated in Figure 10.

Attitudes toward coeliac diagnosis and compliance with a strict gluten-free diet

**Figure 11:**
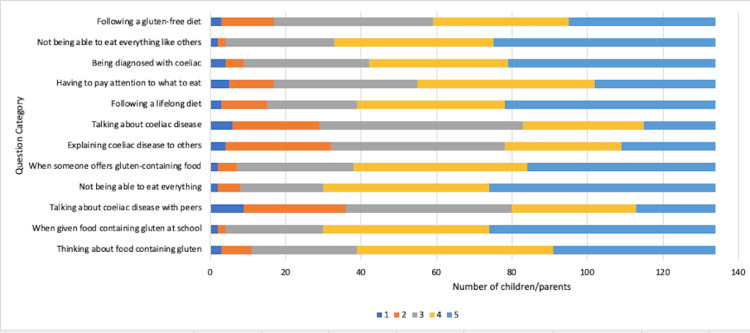
Children’s attitudes towards coeliac disease diagnosis and adherence to a strict gluten-free diet

Attitudes toward CD diagnosis and adhering to a gluten-free diet were themes explored in the second part (CDDUX) of the questionnaire, as seen in Figure 11. Seventeen (12%) children in the cohort felt that following a gluten-free diet is very challenging, with 42 (31%) feeling neutral about the subject; 36 (26.8%) felt confident in talking about their diagnosis while another 44 (32%) felt neutral, with 54 (40%) feeling uncomfortable with talking to others about CD.

Interestingly, 56 (42%) children in the cohort felt that explaining CD to others was difficult; 79 (59%) children felt that choosing the right food items was difficult, and 95 (71%) children felt sad about having to follow a lifelong gluten-free diet.

QoL assessment was compared among several subject groups. When comparing the child vs. parent perspective, no statistically significant difference was found. The same was noted when boys vs. girls, symptomatic vs. asymptomatic, and adolescent (16-18 years) children vs. young (five to seven years) children were compared, as seen in Table 2.

**Table 2:** Comparison between different groups

Kidscreen questionnaire	Asymptomatic vs. symptomatic	Adolescent vs. young child	Male vs. female
Question	P-value	P-value	P-value
Thinking about the last week ... has your child felt fit and well?	0.8728	0.8345	0.262
Thinking about the last week ... has your child been physically active (e.g. running, climbing, biking)?	0.6889	0.4712	0.5752
Thinking about the last week ... has your child been able to run well?	0.5752	0.9362	0.5218
Thinking about the last week ... has your child felt full of energy?	0.7488	0.6889	0.8728
Thinking about last week ... has your child felt that life was enjoyable?	0.8102	0.7488	0.6889
Thinking about last week ... has your child felt pleased that he/she is alive?	0.9362	0.8728	0.4712
Thinking about last week ... has your child felt satisfied with his/her life?	0.8102	0.8728	0.5752
Thinking about last week ... has your child been in a good mood?	0.8102	0.8728	0.5752
Thinking about last week ... has your child felt cheerful?	0.8102	0.7488	0.5218
Thinking about last week ... has your child had fun?	1	0.6889	0.6889
Thinking about last week ... has your child felt that he/she does everything badly?	0.8782	0.6889	0.5218
Thinking about last week ... has your child felt sad?	0.6889	0.7488	0.4233
Thinking about last week ... has your child felt so bad that he/she didn’t want to do anything?	1	0.5752	0.5218
Thinking about last week ... has your child felt that everything in his/her life goes wrong?	0.9362	0.631	0.4712
Thinking about last week ... has your child felt fed up?	0.9362	0.8102	0.298
Thinking about last week ... has your child felt lonely?	0.8102	0.8102	0.4712
Thinking about last week ... has your child felt under pressure?	0.6889	0.5218	0.2298
Thinking about the last week ... has your child been happy with the way he/she is?	0.9362	0.5218	0.8728
Thinking about the last week ... has your child been happy with his/her clothes?	0.7488	0.8728	0.631
Thinking about the last week ... has your child been worried about the way he/she looks?	0.6889	0.5752	0.2002
Thinking about the last week ... has your child felt jealous of the way other girls and boys look?	0.9362	0.8728	0.4233
Thinking about the last week ... has your child wanted to change something about his/her body?	0.8102	0.5752	0.3758
Thinking about last week ... has your child had enough time for him/herself?	0.7488	0.1735	0.5218
Thinking about last week ... has your child been able to do the things that he/she wants to do in his/her free time?	0.8728	0.4712	0.5218
Thinking about last week ... has your child had enough opportunity to be outside?	0.7488	0.6889	0.631
Thinking about last week ... has your child had enough time to meet friends?	0.5752	0.4233	0.5752
Thinking about last week ... has your child been able to choose what to do in his/her free time?	0.6889	0.3785	0.5218
Thinking about the last week ... has your child felt understood by his/her parent(s)?	0.8102	0.8102	0.6889
Thinking about the last week ... has your child felt that his/her parent(s) had enough time for him/her?	0.9362	0.7488	0.5752
Thinking about the last week ... has your child felt that his/her parent(s) treated him/her fairly?	0.9362	0.7488	0.7488
Thinking about the last week ... has your child been able to talk to his/her parent(s) when he/she wanted to?	0.9362	0.4712	0.5752
Thinking about last week ... has your child had enough money to do the same things as his/her friends?	0.8102	0.631	0.8772
Thinking about last week ... has your child felt that he/she had enough money for his/her expenses?	0.8102	1	0.631
Thinking about last week ... does your child feel that he/she has enough money to do things with his/her friends?	0.8102	0.6889	0.5752
Thinking about the last week ... has your child spent time with his/her friends?	1	0.4233	0.4712
Thinking about the last week ... has your child done things with other girls and boys?	0.8782	0.4712	0.631
Thinking about the last week ... has your child had fun with his/her friends?	0.9362	0.4712	0.5218
Thinking about the last week ... has your child and his/her friends helped each other?	0.9362	0.4712	0.5752
Thinking about the last week ... has your child been able to talk about everything with his/her friends?	0.6889	0.8102	0.5752
Thinking about the last week ... has your child been able to rely on his/her friends?	0.9362	0.8102	0.7488
Thinking about the last week ... has your child been happy at school?	0.631	0.4233	0.8728
Thinking about the last week ... has your child got on well at school?	0.9362	0.5752	0.5218
Thinking about the last week ... has your child been satisfied with his/her teachers?	0.6889	0.9362	0.7488
Thinking about the last week ... has your child been able to pay attention?	0.9362	0.7488	0.8102
Thinking about the last week ... has your child enjoyed going to school?	0.6889	0.9362	0.9362
Thinking about the last week ... has your child got along well with his/her teachers?	0.8102	0.8102	0.7488
Thinking about the last week ... has your child been afraid of other girls and boys?	0.6889	0.8728	0.5218
Thinking about the last week ... have other girls and boys made fun of your child?	0.9362	0.7488	0.4712
Thinking about the last week ... have other girls and boys bullied your child?	0.9362	0.2623	0.5218
In general, how would you say your health is?	0.8102	0.3785	0.6889
Thinking about the last week ... have you felt fit and well?	0.9362	0.8728	0.9362
Thinking about the last week ... have you been physically active (e.g. running, climbing, biking)?	0.7488	0.5218	0.5752
Thinking about the last week ... have you been able to run well?	0.8728	0.8728	0.9362
Thinking about the last week ... have you felt full of energy?	0.8102	0.9362	0.9362
Thinking about the last week ... has your life been enjoyable?	0.9362	0.9362	0.5752
Thinking about the last week ... have you felt pleased that you are alive?	0.631	1	0.5218
Thinking about the last week ... have you felt satisfied with your life?	0.9362	1	0.4233
Thinking about the last week ... have you been in a good mood?	0.8102	0.5218	0.7488
Thinking about the last week ... have you felt cheerful?	0.8102	0.6889	0.8728
Thinking about the last week ... have you had fun?	0.8782	0.8728	0.7488
Thinking about the last week ... have you felt that you do everything badly?	1	0.7488	0.3667
Thinking about the last week ... have you felt sad?	0.631	0.9362	0.4233
Thinking about the last week ... have you felt so bad that you didn’t want to do anything?	0.8728	0.631	0.5218
Thinking about the last week ... have you felt that everything in your life goes wrong?	1	0.4712	0.3785
Thinking about the last week ... have you felt fed up?	0.8728	1	0.3785
Thinking about the last week ... have you felt lonely?	0.6889	0.7488	0.6889
Thinking about the last week ... have you felt under pressure?	0.7488	0.8728	0.3367
Thinking about the last week ... have you been happy with the way you are?	0.8102	0.8102	0.4223
Thinking about the last week ... have you been happy with your clothes?	0.7488	0.8102	0.3785
Thinking about the last week ... have you been worried about the way you look?	0.6889	0.8102	0.3367
Thinking about the last week ... have you felt jealous of the way other girls and boys look?	0.5752	0.7488	0.3367
Thinking about the last week ... would you like to change something about your body?	0.7488	0.6889	0.4712
Thinking about the last week ... have you had enough time for yourself?	0.8728	0.298	0.3785
Thinking about the last week ... have you been able to do the things that you want to do in your free time?	0.8728	0.8102	0.631
Thinking about the last week ... have you had enough opportunity to be outside?	0.7488	0.7488	0.4233
Thinking about the last week ... have you had enough time to meet friends?	0.6889	0.5752	0.4233
Thinking about the last week ... have you been able to choose what to do in your free time?	1	0.5218	0.298
Thinking about the last week ... have your parent(s) understood you?	0.8782	0.3667	0.4712
Thinking about the last week ... have you felt loved by your parent(s)?	0.631	0.631	0.3367
Thinking about the last week ... have you been happy at home?	0.8102	0.8728	0.5752
Thinking about the last week ... have your parent(s) had enough time for you?	0.631	0.8728	0.5218
Thinking about the last week ... have your parent(s) treated you fairly?	0.8102	0.8728	0.631
Thinking about the last week ... have you been able to talk to your parent(s) when you wanted to?	0.9362	0.8102	0.298
Thinking about the last week ... have you had enough money to do the same things as your friends?	0.7488	0.8728	0.3367
Thinking about the last week ... have you had enough money for your expenses?	0.8728	0.8102	0.4233
Thinking about the last week ... do you have enough money to do things with your friends?	0.6889	0.4712	0.6889
Thinking about the last week ... have you spent time with your friends?	0.7488	0.7488	0.4712
Thinking about the last week ... have you done things with other girls and boys?	0.631	0.8728	0.6889
Thinking about the last week ... have you had fun with your friends?	0.9362	0.8728	0.8728
Thinking about the last week ... have you and your friends helped each other?	0.6889	0.6889	0.5218
Thinking about the last week ... have you been able to talk about everything with your friends?	0.8728	0.631	0.6889
Thinking about the last week ... have you been able to rely on your friends?	0.6889	0.8728	0.5218
Thinking about the last week ... have you been happy at school?	0.9362	0.8102	0.8728
Thinking about the last week ... have you got on well at school?	0.9362	0.4233	0.7488
Thinking about the last week ... have you been satisfied with your teachers?	0.7488	0.3785	0.4712
Thinking about the last week ... have you been able to pay attention?	0.631	0.7488	0.9362
Thinking about the last week ... have you enjoyed going to school?	0.8728	0.7488	0.9362
Thinking about the last week ... have you got along well with your teachers?	0.9362	0.5218	0.5218
Thinking about the last week ... have you been afraid of other girls and boys?	0.7488	0.6889	0.4233
Thinking about the last week ... have other girls and boys made fun of you?	1	1	0.7488
Thinking about the last week ... have other girls and boys bullied you?	0.9362	0.5218	0.5752
When I think of food containing gluten, I feel ...	0.7448	0.4712	0.4712
When at school I am given food containing gluten, I find it …	0.9362	0.631	0.3785
Talking about my coeliac disease with others my age, I find …	0.6889	0.631	0.2623
Not being able to eat just everything, I find ...	0.8102	0.298	0.631
When someone offers me food that I can’t have, I feel …	1	0.4712	0.3785
When I have to explain to others what coeliac disease is, I feel …	0.6889	0.3785	0.1735
Talking about coeliac disease I find ...	0.8102	0.298	0.3785
Having to follow a lifelong diet, I find …	0.7488	0.3785	0.5218
Having to pay attention to what I eat, I find …	0.8102	0.4233	0.2623
Having coeliac disease is …	0.9362	0.4712	0.3367
Not being able to eat anything I want like other people, I find …	0.8102	0.5218	0.3785
Following a diet for my coeliac disease is ...	0.7488	0.4712	0.298

## Discussion

Child vs. caregiver perspective

The findings of this study show that children in Malta with CD had an overall positive outlook on life and were satisfied with the basic aspects of their life according to the KIDSCREEN-52 questionnaire. This aligns with a study by Mustahlati et al. who reported having CD and following a restrictive diet did not impact the QoL of patients significantly [10]. The perspectives of caregivers and children aligned for most of the variables. Overall good mood and physical health were reported by caregivers and children alike. However, differences between the two groups in terms of the underestimation of the emotional well-being and social relationships of the child in question were noted, even though these were statistically not significant. The QOL of children according to the CDDUX questionnaire revealed a neutral outlook on CD.

A study conducted by Myleus et al. in 2014 assessed QoL in children with CD in Spain. The researchers reported fairly good perspectives toward physical health among both affected children and their caregivers, with the majority describing their health as excellent or very good [11]. This aligns with our study, in which only one child and five caregivers described a negative impact in terms of child health. Similarly, concerns about social life and peer relationships were highlighted in both studies, with lower scores in social and emotional well-being.

Although various studies have shown that there is a tendency for parents to report more negative effects, interestingly, the results in this study highlighted that parents seem to be less aware of the social struggles faced by their children, with more of them expressing positive responses [9,12,13]. An aspect not touched upon in this study is the impact on the caregiver’s QoL due to a CD diagnosis of their child. A child with CD will inevitably affect the entire family and their daily life. The psychological burden of guilt, worry about their child's clinical condition, and anxiety related to possible complications and limited control over their child’s daily activity may be great [14,15]. Additionally, it is important to note that the study does not distinguish between mothers and fathers. A study by Russo et al. noted that mothers reported more lifestyle changes and disease burden than fathers [16].

Financial impact on families

In our study, no particular financial struggles were highlighted for both children and their parents. This is likely explained by the government support offered to these children in the form of monetary vouchers for the purchase of gluten-free products. The various readily available gluten-free options available in both supermarkets and restaurants throughout the islands also contribute to appropriate adherence. This was also reflected in most children reporting feeling well-supported in choosing gluten-free options for their diet.

It is important to note that despite children and their caregivers denying significant financial burdens, a gluten-free diet is more costly than a regular diet. Both supermarket products as well as gluten-free products at restaurants are accompanied by a heftier price tag and often additional charges [16]. Gluten-free products are around 242% more expensive than their gluten-containing counterparts [17]. In the United Kingdom also, monetary vouchers for gluten-free products are available, enabling even low-income families to afford a gluten-free diet. This has recently been discussed due to financial shortenings within the NHS, and the Royal College of Paediatrics and Child Health (RCPCH) has highlighted that low-income families in the UK would not be able to afford a gluten-free diet, especially since one in five children in the UK is currently living in poverty [18]. This further highlights the effectiveness of government monetary vouchers.

The Maltese healthcare system follows a nationalized approach, providing free healthcare for all taxpayers and their children nationwide. Children with CD need lifelong monitoring, in the form of blood investigations, regular clinic visits, imaging, etc. In countries that do not have free healthcare, this can be a financial burden on the family. Having such care freely available on the Maltese islands can be one reason why no significant financial struggle was reported by caregivers and children alike.

Symptomatic vs. asymptomatic patients

When implementing a screening program, the test needs to be highly sensitive and specific. The condition needs to be deemed serious, early diagnosis (even when asymptomatic) can lead to better outcomes and the treatment needs to be available [19]. As mentioned earlier, screening children for CD creates a new cohort of asymptomatic patients. One would speculate that their QoL is impacted negatively by such a diagnosis, as no physical benefits are noticed after starting a strict gluten-free diet. However, our study suggests otherwise. Kinos et al. have noted that when comparing different aspects of QoL, no significant difference was found between asymptomatic and symptomatic children. No difference in adherence to a gluten-free diet was observed. Both groups felt that they had an improvement in their daily life and were satisfied with their diagnosis [20]. A study by Al Nofaie et al. noted that adherence to a gluten-free diet resulted in a better QoL [21]. These are two subject groups we did not compare and it would be interesting to explore them in the future.

Awareness and education

In our study, a large cohort of children felt that explaining their CD diagnosis to others is rather challenging. It would be interesting to know why: whether this is due to a lack of understanding of the disease or ignorance among the population. One could consider that perhaps the child does not understand the meaning of CD diagnosis. A study assessing challenges faced by adolescents with CD diagnosis noted a lack of knowledge about CD and difficulty interpreting food labels [22]. Introducing child-friendly ways to explain the diagnosis in clinics and schools would be an appropriate way to educate both affected and unaffected children and improve adherence. This highlights the importance of increasing awareness and continuous education not only for affected children and their families but also as part of public health campaigns, thereby ensuring adequate support for affected populations outside their homes.

Demographic differences in QoL

No statistically significant difference was noted between different genders and different age groups, despite the presence of various struggles, developments, and lifestyle changes usually associated with adolescence. White et al. did note that adolescents found more difficulty with lifestyle changes and adherence to a gluten-free diet, which is contrary to our results [22]. Our findings noted an increased prevalence of CD in the northern region of the Maltese islands. CD has a genetic and environmental component, which could explain why it is more prevalent in the north of Malta; however, this would have to be investigated further.

Limitations

This study has a few limitations. Computer literacy, access to the internet, and access to email were necessary for participation and thus could have inadvertently led to the exclusion of low socioeconomic status families and inaccurate/missing responses from young children due to difficulty in answering questions themselves or using the appropriate software to do so. Excluding families from low socioeconomic backgrounds could have skewed data towards more positive answers on financial burden or emotional well-being. The questionnaire was only available in the English language, as the CDDUX and KIDSCREEN have not been translated into the native Maltese language. Participants might have struggled with understanding the questionnaire and non-English speakers would have been automatically excluded from participation. Both the KIDSCREEN and CDDUX are generic questionnaires to be used for 8- to 18-year-olds; however, in our cohort of patients, children aged five to seven years were also included. This patient group would have needed assistance from their caregiver to answer apart from facing difficulty with certain questions such as those relating to financial matters, leading to response bias.

Although caregivers were advised that children should respond independently, this could not be ensured as the questionnaire was answered at home, also potentially contributing to response bias. Data on symptomatology were not available for all included participants, which could have skewed results. This could be avoided by controlling the environment in which patients fill out the survey, allowing for monitoring of correct application and independent results. Although ours was a nationwide cohort, the small sample size needs to be highlighted. The study was carried out during the COVID-19 pandemic which could have affected results; hence, repeating this study would be beneficial to assess for bias. Certain measures can be undertaken to reduce limitations in future studies. KIDSCREEN and CDDUX in the Maltese language would reduce cultural and language bias. Also, conducting the survey during clinic visits would allow assistance to be available, which would ensure a larger cohort of patients from all backgrounds. 

The strength of this study mainly lies in the fact that it is the first population-based study of its kind among the Maltese population following a period of nationwide screening for CD. This has allowed us to recruit a mixed cohort representative of the population, with symptomatic and asymptomatic individuals across both genders and all pediatric age groups. The use of validated tools - KIDSCREEN and CDDUX - lends significance to the study. However, it is important to highlight that such validated tools may be interpreted differently in different cultures, which is another potential limitation of the study.

## Conclusions

Based on our findings, the QoL of children with CD in Malta is satisfactory overall. The perspectives of parents and children on their CD diagnosis and its impact on QoL mostly align, with differences mainly noted in awareness of emotional and social struggles faced by diagnosed children. The study also highlights that despite overall good health, providing adequate psychological support for these children and their families is important, together with raising further public awareness about this condition. Family-centered interventions can enhance QoL within the family unit. It is important to continue to put in efforts to better understand the QoL of children with CD to strive towards improved adherence and health outcomes.
